# Lanthanide Chelates as a Tool in Nucleic Acid Chemistry

**DOI:** 10.1155/MBD.1994.201

**Published:** 1994

**Authors:** Veli-Matti Mukkala, Harri Takalo, Päivi Liitti, Jouko Kankare, Satu Kuusela, Harri Lönnberg

**Affiliations:** 1 Department of Chemistry University of Turku Turku FIN-20500 Finland; 2 Wallac Oy P.O. Box 10 Turku FIN-20101 Finland

## Abstract

The potentiality of lanthanide chelates as photoluminescent markers and cleaving agents of nucleic acids is discussed, the main emphasis being on the chelates derived from aromatic nitrogen bases.


### LANTHANIDE CHELATES AS A TOOL IN NUCLEIC ACID CHEMISTRY

Veli-Matti Mukkala[1,2], Harri Takalo[2], Päivi Liitti[2], Jouko Kankare[1], Satu Kuusela[1] and Harri Lönnberg*[1]

[1] Department of Chemistry, University of Turku, FIN-20500 Turku, Finland
[2] Wallac Oy, P.O. Box 10, FIN-20101 Turku, Finland

*Abstract:* The potentiality of lanthanide chelates as photoluminescent markers and cleaving agents of nucleic acids is discussed, the main emphasis being on the chelates derived from aromatic nitrogen bases.

**Introduction**

Lanthanide(III) ions and their organic chelates constitute a versatile tool in nucleic acid chemistry and molecular biology. On one hand, they may be used as luminescent markers that enable sensitive detection of nucleic acids in DNA-hybridization assays[1-4] or in *in situ* fluorescence imaging.[5] On the other hand, lanthanide(III) ions and some of their chelates exhibit considerable catalytic activity towards hydrolysis of internucleosidic phosphodiester bonds of RNA, [6-8] a property that may be utilized in developing chemically reactive antisense oligonucleotides for selective inhibition of gene expression. Furthermore, lanthanide chelates have found usage in NMR imaging,[9] in determination of the multiplicity of metal ion binding sites in biopolymers,[10] and in NMR spectroscopy as shift reagents.[11] The present article is focused on the potentiality of lanthanide(III) chelates as photoluminescent markers and as cleaving agents of ribonucleic acids.

**Lanthanide Chelates as Photoluminescent Markers**

Lanthanide(III) ions as such are only weakly luminescent in aqueous solution, because their molar absorptivity is low and the excited states are effectively quenched by solvent molecules with weak light emission. Coordinated aromatic ligands may greatly enhance the luminescence by absorbing energy and transferring it to the central ion, and by extruding water molecules from the inner coordination sphere of the lanthanide(III) ion. With Eu(III) and Tb(III) chelates, the energy transfer from the excited single state of the ligand to its triplet state and further to the metal ion is effective, as shown by the fact that the strongest emission of these chelates is the long-lived luminescence emanating from the metal ion.[12] With the chelates of the other lanthanide ions, the excited ligand may return to the ground state either with concomitant emission of a prompt ligand luminescence, or without light emission. Eu(III) and Tb(III) chelates also have some other properties that make their usage as luminescent markers advantageous:[13] (i) the difference between the wavelength of excitation and main emission is usually large [> 270 nm with Eu(III) and > 200 nm with Tb(III)], (ii) the luminescence life-time is long [of the order of 1 ms compared to 10 ns of the normal background], (iii) the emission signals are narrow [half-width typically < 10 nm], and (iv) the concentration quenching is small.

The use of photoluminescent chelates of Eu(III) and Tb(III) as markers would undoubtedly offer the most straightforward strategy for the luminescence based hybridization assays. Moreover, they would enable *in situ* detection of labelled nucleic acids. In spite of these marked advantages, only few assays exploiting photoluminescent chelates have been reported.[3,14] Usually the techniques thus far applied utilize non-luminescent chelates, and the luminescence is then generated in a separate step that follows the actual bioaffinity reaction. Evidently it has been difficult to achieve with stable luminescent chelates under the conditions of hybridization assay an equally high luminescence yield as with a separate luminescence enhancement method. The chelate should exhibit the following features: (i) high chemical, photochemical and kinetic stability, (ii) long wavelength of excitation (preferrably > 330 nm), (iii) high absorptivity at the wavelength of excitation, (iv) efficient energy transfer from the ligand to the central ion, (v) long luminescence life-time, (vi) good solubility in water and (vi) minor influence on the binding behaviour of the biomolecule. The attempts to prepare such chelates are described below.

The photoluminescent lanthanide chelates, aimed at fulfilling the requirements listed above, usually consist of 4 structural moieties: (i) an Eu(III) or Tb(III) ion [sometimes Sm(III) or Dy(III)], (ii) an aromatic structure that absorbs the excitation energy and transfers it to the lanthanide(III) ion, (iii) additional chelating groups that ensure high kinetic stability, and (iv) a reactive group used to attach the chelate to the biomolecule. Moreover, there may be a tether group between the reactive group and the absorbing aromatic moiety. An example is given in Fig. I.

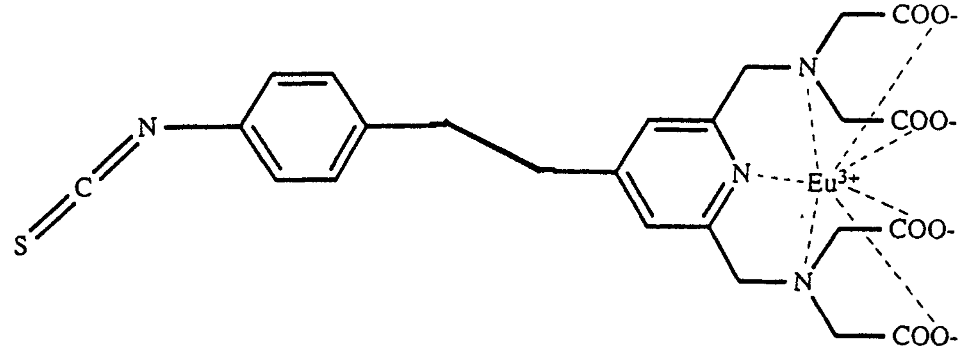

| Reactive group for coupling | Tether group | Excitation energy absorbing moiety | Chelating groups and lanthanide ion |
| --- | --- | --- | --- |

Fig. I: An example of a general structure of a photoluminescent lanthanide chelate suitable for labelling of biomolecules.

As mentioned above, the aromatic ligand should absorb the excitation energy at a long wavelength, preferably longer than 330 nm, since then the choice of materials of the instrument is more flexible and the background luminescence is less significant. The higher absorption wavelength usually means a more conjugated system and hence a lower energy level of the triplet state. The triplet state energy level of the ligand must, however, remain higher than the resonance level of the lanthanide ion. Otherwise the intrachelate energy transfer to the central ion cannot take place. This energy transfer is mediated by a heteroatom that is part of the conjugated system and is simultaneously coordinated to the lanthanide ion. The best alternatives for the heteroatom are thus nitrogen and oxygen, since they are, as relatively hard Lewis bases, preferred by the lanthanide(III) ions.

To find an heteroaromatic structure that efficiently absorbs the excitation energy at a long wavelength and transfers it to the coordinated lanthanide ion, the luminescence properties of the Eu(III) and Tb(III) chelates of numerous aromatic nitrogen bases bearing two 2,2'-(methylenenitrilo)-*bis*(acetic acid) groups have been compared.[15] As seen from the data in Table I, 2,2'-bipyridine (**5**) and 2,2':6',2"-terpyridine derivatives (**12**) exhibited, as both the Eu(III) and Tb(III) chelates, the highest luminescence yields among the ligands studied. Accordingly, they may be regarded as potent energy absorbing moieties for the further development of photoluminescent chelates. With both ligands (**5**,**12**), the excitation takes place at a reasonably high wavelength region, and the life-time of luminescence is long. Pyridine,[4,5] and 1,10-phenanthroline[17-19] based photoluminescent chelates

**Table I: Relative luminescence yields (log $R$),[a] excitation maxima ($\lambda_{exc}$) and luminescence life-times ($\tau$) of the Eu(III) and Tb(III) chelates of ligands derived from aromatic nitrogen bases.[15]**

| Ligand | Parent heterocycle | Eu(III) chelate | | | Tb(III) chelate | | |
|---|---|---|---|---|---|---|---|
| | | log $R$ | $\lambda_{exc}$/nm | $\tau$/ms | log $R$ | $\lambda_{exc}$/nm | $\tau$/ms |
| **1** | Pyridine | 4.83 | 265 | 0.40 | 4.87 | 267 | 1.3 |
| **2** | Isoquinoline | 4.44 | 324 | 0.38 | *b* | | |
| **3** | Benz[*f*]isoquinoline | 4.41 | 355 | 0.38 | 2.35 | 355 | 0.02 |
| **4** | Acridine | *b* | | | *b* | | |
| **5** | 2,2'-Bipyridine | 5.50 | 307 | 0.59 | 5.27 | 307 | 1.2 |
| **6** | 3,3'-Biisoquinoline | 5.33 | 330 | 0.36 | *b* | | |
| **7** | Di(pyrid-2-yl)ketone | 4.61 | 272 | *c* | 4.51 | 272 | *d* |
| **8** | 2,2'-Bipyrimidine | 5.17 | 250 | 0.51 | *b* | | |
| **9** | 4,4'-Bipyrimidine | 5.08 | 290 | 0.56 | 2.27 | 284 | *e* |
| **10** | 1,8-Naphthyridine | 4.65 | 312 | 0.32 | 4.50 | 312 | 0.98 |
| **11** | 1,10-Phenanthroline | 5.26 | 272 | *f* | 4.71 | 272 | 0.68 |
| **12** | 2,2':6',2"-Terpyridine | 5.94 | 333 | *g* | 5.64 | 333 | 1.3 |

[a]$R = (c_{Ln}/c_{ch})(k_{Ln}/k_{ch})(I_{ch}/I_{Ln})$, where $c_{ch}$, $k_{ch}$ and $I_{ch}$ denote the concentration, emission decay constant and luminescence intensity of the lanthanide chelate, and $c_{Ln}$, $k_{Ln}$ and $I_{Ln}$ are the corresponding quantities of the free lanthanide ion.[16] When the emission decay is multiexponential, $I_{ch}/k_{ch}$ is replaced by $\Sigma I_{ch,i}/k_{ch,i}$. [b]Luminescence too weak to be measured. [c]Biexponential decay with $\tau$ = 0.25 and 0.98 ms. [d]Biexponential decay with $\tau$ = 0.24 and 1.9 ms. [e]Biexponential decay with $\tau$ = 0.17 and 0.63 ms. [f]Biexponential decay with $\tau$ = 0.23 and 0.51 ms. [g]Biexponential decay with $\tau$ = 0.19 and 1.4 ms.

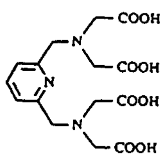

**1**

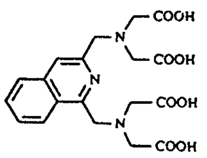

**2**

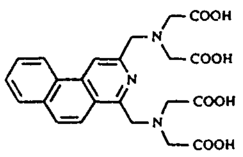

**3**

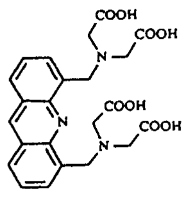

**4**

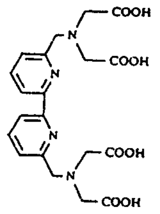

**5**

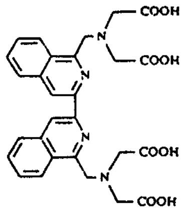

**6**

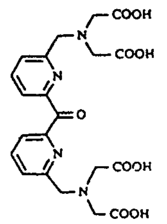

**7**

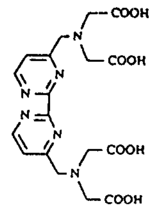

**8**

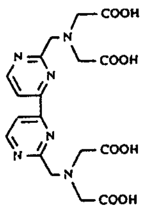

**9**

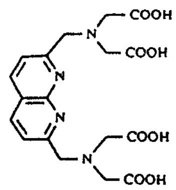

**10**

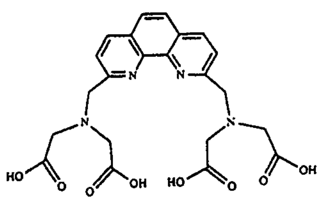

**11**

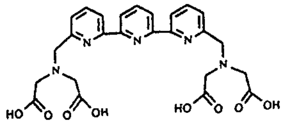

**12**

have also been used in bioaffinity assays, but their luminescence properties appear inferior to those of bipyridine and terpyridine chelates. It is also interesting to note that the luminescence yield cannot be increased by simply increasing the conjugation of the energy absorbing moiety. Acridine chelates, for example, exhibit a very low luminescence, in spite of their extensive conjugation. Evidently, the increased conjugation has led to a decreased triplet state energy, and hence the energy transfer to the central ion is impeded.

A possible way to improve the luminescence properties of the lanthanide chelates of **5** and **12** is to insert substituents in their energy absorbing moiety. As seen from Table II, this approach seems to be of limited importance.[16] Most of the substituents on either one or both of the pyridine rings of **5** reduce the luminescence yield, and no simple correlation between the magnitude of the effect and the polar properties of the substituent appears to exist. The most favourable substitution among those studied is 4,4'-dimethylation that slightly enhances the luminescence yield of both the Eu(III) and Tb(III) chelates, leaving the excitation wavelength and luminescence life-time practically unchanged.

Table II: The effect of substituents on the relative luminescence yields (log *R*),[a] excitation maxima ($\lambda_{exc}$) and luminescence life-times ($\tau$) of the Eu(III) and Tb(III) chelates of 2,2',2'',2'''-[(2,2'-bipyridine-6,6'-diyl)*bis*(methylenenitrilo)]*tetrakis*(acetic acid), **5**.[16]

| Substituents on **5** | Eu(III) chelate | | | Tb(III) chelate | | |
|---|---|---|---|---|---|---|
| | log *R* | $\lambda_{exc}$/nm | $\tau$/ms | log *R* | $\lambda_{exc}$/nm | $\tau$/ms |
| None | 5.50 | 307 | 0.59 | 5.27 | 307 | 1.2 |
| 4,4'-Dimethyl | 5.61 | 310 | 0.59 | 5.51 | 308 | 1.5 |
| 4-Nitro | 4.12 | 328 | 0.53 | 2.94 | 310 | 1.4 |
| 4,4'-Dinitro | 4.14 | 338 | 0.53 | *b* | | |
| 4-Ethoxy | 5.33 | 298 | 0.58 | 5.31 | 299 | 1.6 |
| 4,4'-Diethoxy | 5.11 | 290 | 0.56 | 5.13 | 287 | 1.5 |
| 4-Bromo | 5.36 | 315 | 0.57 | 5.12 | 310 | 1.0 |
| 4,4'-Dibromo | 5.31 | 310 | 0.54 | 5.02 | 310 | 0.75 |
| 5-Bromo | 4.16 | 320 | 0.56 | 4.50 | 320 | *c* |
| 4-Amino | 4.42 | 290 | 1.72 | 4.62 | 294 | 1.5 |
| 3,3'-Dihydroxy | 3.91 | 340 | 0.38 | *b* | | |
| 3,3'-*Bis*(ethoxycarbonyl) | 4.31 | 275 | 0.56 | *b* | | |
| 4,4'-Dicarboxy | 4.42 | 325 | 0.58 | 3.54 | 325 | 0.63 |
| 3,3'-Dicarboxy | 4.90 | 283 | 0.57 | 2.23 | 280 | *d* |
| 3,3'-Dibenzoxy | 5.36 | 292 | 0.54 | 2.83 | 290 | *e* |
| 4,4'-Diphenyl | 5.52 | 325 | 0.58 | 5.18 | 325 | 0.89 |
| 4,4'-*Bis*(4-methoxyphenyl) | 5.57 | 325 | 0.56 | 4.84 | 325 | 0.33 |
| 4,4'-*Bis*(2-furyl) | 5.32 | 330 | 0.57 | 2.10 | 320 | 0.67 |
| 4,4'-Distyryl | 2.95 | 315 | 0.57 | 2.63 | 313 | 0.90 |
| *N,N'*-Dioxide | 4.72 | 280 | *f* | 3.74 | 280 | *g* |

[a]See footnote *a* in Table I. [b]Luminescence too weak to be measured. [c]Biexponential decay with $\tau$ = 0.10 and 0.73 ms. [d]Biexponential decay with $\tau$ = 0.18 and 1.6 ms. [e]Biexponential decay with $\tau$ = 0.10 and 0.63 ms. [f]Biexponential decay with $\tau$ = 0.20 and 0.74 ms. [g]Biexponential decay with $\tau$ = 0.21 and 0.90 ms.

An alternative manner to affect the luminescence properties of **5** and **12** is the replacement of one or two of their pyridine rings with five-membered heteroaromatic rings. Because the ring angles are in five-membered rings smaller than in pyridine, the three dimensional structure or these chelates differs from that of the parent chelates. Moreover, some five-membered heterocycles, such as 1,2,4-

triazole, may loose a proton upon the chelate formation, and hence the total charge of the chelate is changed. As seen from Table III, the chelates of the terpyridine analogues containing one or two five-membered structural units do not, however, exhibit markedly enhanced luminescence yields compared to the chelates of **12**. Only the Tb(III) chelate derived from 3,5-di(pyrid-2-yl)-1,2,4-triazole (**16**) is somewhat more strongly luminescent than the corresponding terpyridine chelate. With this chelate the luminescence life-time is exceptionally long, more than double compared to that of the Tb(III) chelate of **12**. Evidently the water molecules are unusually effectively extruded from the inner coordination sphere of the Tb(III) ion. Interestingly, the luminescence yield of the Eu(III) chelate is, in turn, smaller than that of the terpyridine chelate.

Table III: Relative luminescence yields (log *R*),[a] excitation maxima ($\lambda_{exc}$) and luminescence life-times ($\tau$) of the Eu(III) and Tb(III) chelates of some structural analogues of 2,2',2'',2'''-[(2,2':6',2''-terpyridine-6,6''-diyl)*bis*(methylenenitrilo)]*tetrakis*(acetic acid) (**12**).[20]

| Ligand | Parent heterocycle | Eu(III) chelate | | | Tb(III) chelate | | |
|---|---|---|---|---|---|---|---|
| | | log *R* | $\lambda_{exc}$/nm | $\tau$/ms | log *R* | $\lambda_{exc}$/nm | $\tau$/ms |
| **12** | 2,2':6',2''-Terpyridine | 5.94 | 333 | *b* | 5.64 | 333 | 1.3 |
| **13** | 2,6-*Bis*(thiazol-2-yl)pyridine | 5.73 | 340 | *c* | *d* | | |
| **14** | 2,6-*Bis*(thiazol-4-yl)pyridine | 5.05 | 314 | *e* | 4.60 | 316 | 0.86 |
| **15** | 2,4-Di(pyrid-2-yl)thiazole | 5.79 | 330 | 0.89 | *d* | | |
| **16** | 3,5-Di(pyrid-2-yl)-1,2,4-triazole | 5.13 | 280 | *f* | 5.76 | 310 | 2.9 |

[a]See footnote *a* in Table I. [b]Biexponential decay with $\tau$ = 0.20 and 1.4 ms. [c]Biexponential decay with $\tau$ = 0.20 and 1.4 ms. [d]Luminescence too weak to be measured. [e]Biexponential decay with $\tau$ = 0.25 and 1.2 ms. [f]Biexponential decay with $\tau$ = 0.22 and 0.91 ms.

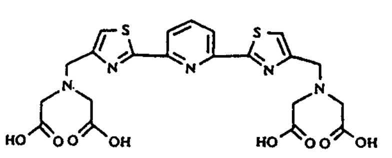

**13**

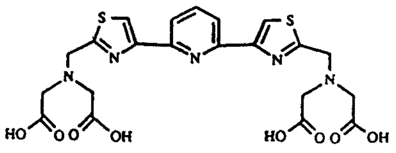

**14**

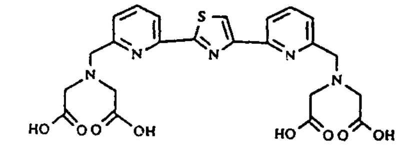

**15**

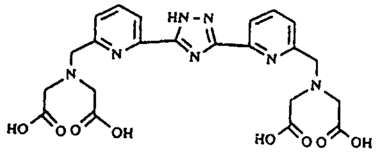

**16**

In luminescent chelates, the chelating groups also affect the luminescence properties. They extrude water molecules from the inner coordination sphere of the central ion, and hence may be expected to prolong the luminescence life-time and increase the luminescence yield. The chelating groups may also have an influence on the energy level of the triplet state; this is in particular the case with chelating groups that conjugate with the energy absorbing moiety. Table IV summarizes the effects that various chelating groups exert on the luminescence properties of the Eu(III) and Tb(III) chelates of 2,2'-bipyridine ligands functionalized with these groups at C6 and C6'. The excitation wavelength shows only minor variation from one compound to another, correlating closely with the absorption spectra of the chelates. Upon chelate formation, the absorption maxima are invariably shifted to a higher wavelength by 15 to 30 nm, most likely owing to polarization of the 2,2'-bipyridine moiety and its conformational change from *s-trans* to *s-cis* with concomitant planarization. As seen, the effect of chelating groups on the luminescence yield is generally only a moderate one. However, carboxy [with Tb(III)], methylenephosphonic acid and, in particular, 2,2'-(carbonylnitrilo)*bis*(acetic acid) groups tend to decrease the luminescence yield. Possibly conjugation of the carbonyl function with the bipyridine system has an unfavourable effect on the triplet state energy level. It is worth noting that the widely used 2,2'-(methylenenitrilo)*bis*(acetic acid) and its phosphonate analogue, 2,2'-(methylenenitrilo)*bis*(methylenephosphonic acid), give rise to the highest luminescence yields among the chelating groups studied.

Table IV: The effect of chelating groups on the relative luminescence yields (log *R*),[a] excitation maxima ($\lambda_{exc}$) and luminescence life-times ($\tau$) of the Eu(III) and Tb(III) chelates of 6,6'-functionalized 2,2'-bipyridine ligands.[21]

| Chelating groups at C6 and C6' of 2,2'-bipyridine | Eu(III) chelate log *R* | $\lambda_{exc}$/nm | $\tau$/ms | Tb(III) chelate log *R* | $\lambda_{exc}$/nm | $\tau$/ms |
|---|---|---|---|---|---|---|
| -COOH | 5.58 | 314 | *b* | 4.74 | 312 | *c* |
| $-CH_2N(CH_2COOH)_2$ | 5.50 | 307 | 0.59 | 5.27 | 307 | 1.2 |
| $-CH_2NCH(COOH)(CH_2)_3CH(COOH)$ | 5.42 | 310 | 0.67 | 5.23 | 308 | 0.96 |
| $-CH_2N(CH_2COOH)CH(COOH)(CH_2)_4OH$ | *d* | | | 5.02 | 308 | 1.2 |
| $-CH_2N(CH_2COOH)CH(COOH)CH_2COOH$ | 5.30 | 306 | 0.69 | 5.43 | 306 | 1.6 |
| $-C(O)N(CH_2COOH)_2$ | *e* | | | 3.43 | 310 | 0.74 |
| $-CH_2PO_3H_2$ | 4.81 | 315 | *f* | 4.74 | 315 | 0.50 |
| $-CH_2N(CH_2PO_3H_2)_2$ | 5.67 | 318 | *g* | 5.81 | 314 | *h* |

[a]See footnote *a* in Table I. [b]Biexponential decay with $\tau$ = 0.19 and 0.77 ms. [c]Biexponential decay with $\tau$ = 0.17 and 0.49 ms. [d]Not determined. [e]Luminescence too weak to be measured. [f]Biexponential decay with $\tau$ = 0.16 and 0.29 ms. [g]Biexponential decay with $\tau$ = 0.21 and 1.3 ms. [h]Biexponential decay with $\tau$ = 0.13 and 0.48 ms.

Previously,[22,23] it has been shown that photoluminescent chelates, exhibiting luminescence yields comparable to that of **5** and **12**, may be obtained from 2,2'-bipyridine by closing the lanthanide ion inside a cryptate structure consisting of 3 6,6'-dimethylene-2,2'-bipyridine bridges between two nitrogen atoms. Comparison of the decay constants in $H_2O$ and $D_2O$ has suggested that the Eu(III) chelate contains from 2 to 3 water molecules and the Tb(III) chelate less than one molecule in the inner coordination sphere. It has also been shown that replacing one of the bridging groups by 2 nonbridging 6-methylene-2,2'-bipyrimidine groups,[24] or oxidizing one of the bipyridine units to its *N,N'*-dioxide,[25] completely protects the lanthanide ion from the interaction with water. The fact that the luminescence life-times of the chelates of **5** and **12** are comparable to those of these cryptate chelates suggests that binding of a lanthanide ion to 2 2,2'-(methylenenitrilo)*bis*(acetic acid) groups, in addition to 2 or 3 pyridine nitrogens, is sufficient to shield it quite effectively from the solvent molecules.

Attachment of a lanthanide chelate to a biomolecule may considerably change its luminescence properties. This is clearly seen from Table V that lists the excitation maxima, luminescence life-times and luminescence yields for the Eu(III) and Tb(III) chelates derived from terpyridine (**12**), its 4'-substituted derivatives and their protein conjugates.[26] Coupling of the Eu(III) chelates to proteins *via* an isothiocyanate or 4,6-dichloro-1,3,5-triazin-2-ylamino function does not markedly reduce their luminescence. By contrast, the Tb(III) chelates appear to be in this respect much more sensitive. For example, insertion of a 2-(4-aminophenyl)ethyl substituent as a tether group at C4' of **12** reduces the luminescence yield to about 1 % of that of the unsubstituted chelate. This quenching is partly recovered upon further modification of the amino group to isothiocyanato or 4,6-dichloro-1,3,5-triazin-2-ylamino groups and reacting them with proteins. Changes in the triplet state energy may possibly result in an energy leakage from Tb(III) back to the ligand. The luminescence life-times of the Eu(III) and Tb(III) chelates also behave in a very different manner. The life-times of all Eu(III) chelates remain practically constant, irrespective of the molecular environment. With Tb(III) chelates, the life-time is considerably decreased upon formation of a protein conjugate. Comparative measurements in $D_2O$ gave parallel results, and hence the short life-times do not primarily result from hydroxyl quenching, but some other mechanism must operate.

Table V: The luminescence yields ($\epsilon \times \Phi$), excitation maxima ($\lambda_{exc}$) and luminescence life-times ($\tau$) of the Eu(III) and Tb(III) chelates of 4'-substituted 2,2',2",2"'-[(2,2':6',2"-terpyridine-6,6"-diyl)*bis*(methylenenitrilo)]*tetrakis*(acetic acids) and their protein[a] conjugates.[26]

| Substituent at C4' | Eu(III) chelate | | | Tb(III) chelate | | |
|---|---|---|---|---|---|---|
| | $\epsilon \times \Phi$ | $\lambda_{exc}$/nm | $\tau$/ms | $\epsilon \times \Phi$ | $\lambda_{exc}$/nm | $\tau$/ms |
| *Uncoupled chelates* | | | | | | |
| None | 2100 | 334 | 1.3 | 3800 | 333 | 1.1 |
| Phenyl | 1970 | 293 | 1.2 | 1900 | 293 | 0.53 |
| 2-(4-Aminophenyl)ethyl | 220 | 331 | 1.2 | 53 | 332 | 0.70 |
| *Protein conjugates* | | | | | | |
| 4-Isothiocyanatophenyl | 2100 | 340 | 1.4 | *b* | | |
| 4-(4,6-Dichloro-1,3,5-triazin-2-ylamino)phenyl | 2600 | 340 | 1.6 | *b* | | |
| 3-Isothiocyanatophenyl | 1300 | 295 | 1.5 | *b* | | |
| 3-(4,6-Dichloro-1,3,5-triazin-2-ylamino)phenyl | 600 | 330 | 1.5 | *b* | | |
| 4-(3-Isothiocyanatobenzyloxy)phenyl | 2500 | 340 | 1.5 | 190 | 334 | 0.05 |
| 4-[3-(4,6-Dichloro-1,3,5-triazin-2-ylamino)-benzyloxy]phenyl | 1800 | 333 | 1.5 | 190 | 333 | 0.11 |
| 3-(3-Isothiocyanatobenzyloxy)phenyl | 790 | 297 | 1.2 | 35 | 300 | 0.03 |
| 3-[3-(4,6-Dichloro-1,3,5-triazin-2-ylamino)-benzyloxy]phenyl | 2600 | 296 | 1.4 | 190 | 295 | 0.06 |
| 2-(4-Isothiocyanatophenyl)ethyl | 680 | 332 | 1.5 | 130 | 332 | 0.09 |
| 2-[4-(4,6-Dichloro-1,3,5-triazin-2-ylamino)-phenyl]ethyl | 840 | 332 | 1.5 | 490 | 331 | 0.45 |

[a]Rabbit anti-mouse IgG. [b]Luminescence too weak to be measured.

Luminescent chelates still need optimization, although major improvements cannot be taken as granted. In many chelates, the energy transfer from the ligand to the lanthanide ion is already very high. Increasing the absorptivity of the ligand may, in turn, lower the triplet state energy too much, and hence retard the energy flow to the lanthanide ion. The luminescence life-time of the Eu(III) chelates has already reached its limit, but with Tb(III) further optimization is still needed. In particular, the quenching that coupling to biomolecules results in should be minimized. Because lanthanide chelates do not markedly suffer from concentration quenching, chelate clusters could be synthesized and used instead of individual chelates. In addition to chelates based on aromatic nitrogen bases, some other types of ligands have proved to be interesting, though still inadequately studied. These include, above all, $\beta$-diketones[27] and ligands containing several phenolic units.[28-31]

### Lanthanide Ions and Chelates as Cleaving Agents

It has been recently shown that lanthanide ions catalyze the phosphoester hydrolysis of nucleoside monophosphates,[8] nucleoside cyclic monophosphates,[32,33] dinucleoside monophosphates[7,8,34] and polyribonucleotides[6] more efficiently than other metal ions. In particular, the hydrolysis of cyclic phosphates is extremely susceptible to the presence of lanthanide ions; the hydrolysis of uridine 2',3'-cyclic monophosphate, for example, is accelerated by a factor of $10^8$ at $[Eu^{3+}]$ = 10 mM under conditions where the metal ion starts to precipitate (pH 7.2 at 303.2 K).[32] It is also important to note that lanthanide ions promote only the hydrolysis of nucleoside phosphoesters, not the intramolecular transesterification of the phosphoric acid residue from O3' to O2'.[8] This is of major importance in attempting to develop metal based catalysts for the cleavage of internucleosidic phosphodiester bonds of RNA. Most likely the mechanism depicted in Scheme I is followed in principle: the metal ion is coordinated to the anionic phosphate group and its hydroxo ligand acts as a general acid-base catalyst, deprotonating the attacking 2'-OH and protonating the departing 5'-O$^-$. The pseudo-

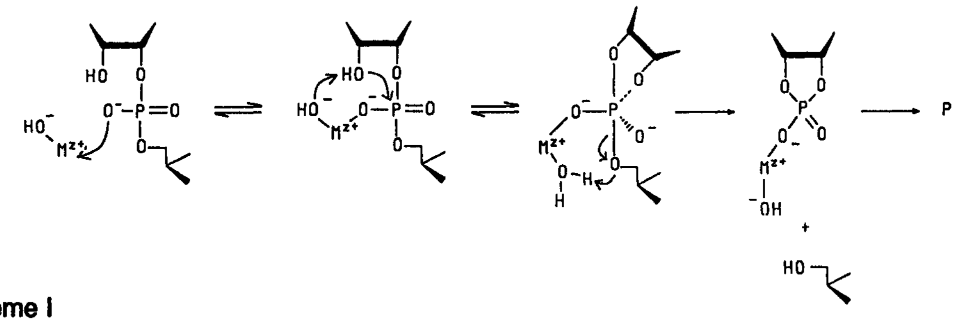

Scheme I

rotation of the phosphorane intermediate and the subsequent cleavage of the P-O3' bond is not accelerated to a comparable extent, and hence the phosphate migration that in the absence of metal ions occurs even faster than the hydrolysis of the phosphodiester bond, becomes a minor side reaction.[35] However, the details of the mechanism of the lanthanide ion action are obscure. The lanthanide-ion-catalyzed phosphoester hydrolyses typically exhibit curved pH-rate profiles, the reaction order in the hydroxide ion concentration being increased from 1 to almost 3 on approaching the pH where the precipitation of the metal ion starts (Fig. II). Interestingly,

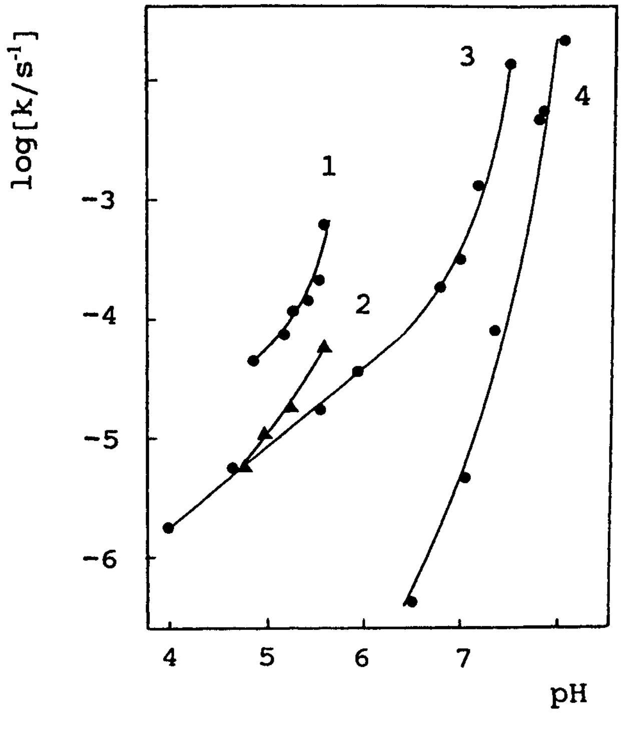

Fig. II: Effect of pH on the lanthanide-ion-catalyzed hydrolysis of nucleoside phosphoesters: (1) uridylyl(3',5')uridine + Gd(III) at 363.2 K,[8] (2) uridine 3'-monophosphate + Gd(III) at 363.2 K,[8] uridine 2',3'-cyclic monophosphate + Eu(III) at 303.2 K,[32] adenylyl(3',5')adenosine + Gd(III) at 293.2 K.[36]

precipitation of the lanthanide ion in the course of a kinetic run considerably accelerated the phosphoester hydrolysis (Fig. III).[36] By contrast, the lanthanide hydroxide gel prepared beforehand exhibited a lower catalytic activity. These findings suggest that the catalytically active species is not a lanthanide aquo ion, but a polynuclear hydroxo complex resembling the lanthanide hydroxide gel. With Ce(III) ion, the catalytically active species has been suggested to be $[Ce_3(OH)_5]^{4+}$.[33]

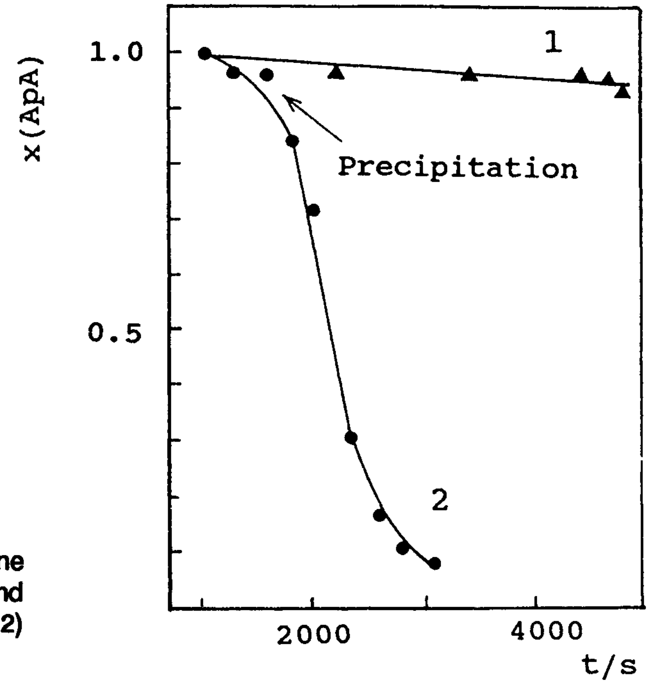

Fig. III: Hydrolysis of adenylyl(3',5')adenosine in 10 mM aqueous $Gd(NO_3)_3$ at pH 7.4 and 293.2 K, when Gd(III) precipitated (curve 2) and when it remained in solution (curve 1).

Kinetically stable chelate is a prerequisite for the development of lanthanide ion based catalysts that would cleave RNA in a sequence-specific manner. Water (and hydroxide ions) have such a high affinity to lanthanide ions that in aqueous solution only negatively charged oxygens may replace the aquo ligands. Binding of the lanthanide ions to negatively charged ligands, however, markedly reduces their catalytic activity.[32] That is why the observation of Morrow *et al.*[6] that shows La(III) ion to retain a considerable part of its catalytic activity on binding to a neutral 18-membered macrocyclic Schiff base, 17, is noteworthy, and it may be regarded as the first step towards sequence selective hydrolysis (not cleavage by oxidation). However, this chelate does not yet fulfil all the requirements

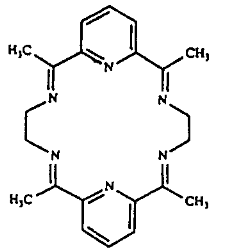

17

of a good cleaving agent. According to our recent studies,[36] it is not hydrolytically stable enough, but decomposes concurrent with the hydrolysis of 3',5'-UpU. In addition, the Schiff base ligand, 17, is quite unstable in the absence of the lanthanide ion, and usually it is synthesized using the lanthanide ion as a template. Attachment of this type of a chelate to an oligonucleotide probe, or any other molecule that recognizes the base sequence of RNA, may prove difficult. In spite of these problems, lanthanide chelates still comprise, perhaps together with macrocyclic triaza complexes of Zn(II), the most potent class of compounds for the further development of artificial RNA-nucleases.

**Attachment of Lanthanide Chelates to Oligonucleotides**

To be used as luminescence markers of cleaving agents, the lanthanide chelates must be attached to oligonucleotides. Usually this is done by allowing a reactive group of the chelate, such as isothiocyanate or 4,6-dichloro-1,3,5-triazin-2-ylamino group, to react with an amino or thiol functions tethered to the oligonucleotide. The chelates have conventionally been attached to the 5'-end of the oligonucleotide probes by using structurally modified building blocks.[37] We have recently introduced some additional methods, both chemical and enzymatic, for labelling the 3'-terminal[38] or nonterminal nucleosides[39] at the sugar moiety. Together with more sensitive markers and more efficient cleaving agents these methods may further the versatility of lanthanide chelates as a tool in nucleic acid chemistry.

*Acknowledgments.* Financial support from the Academy of Finland, the Research Council for Natural Sciences, is gratefully acknowledged.

**References**

1. Hurskainen, P.; Dahlen, P.; Ylikoski, J.; Kwiatkowski, M.; Siitari, H.; Lövgren, T. *Nucleic Acids Res.* **1991**, *19*, 1057.
2. Oser, A.; Valet, G. *Angew. Chem. Int. Ed.* **1990**, *29*, 1167.
3. Prat, O.; Lopez, E.; Mathis, G. *Anal. Biochem.* **1991**, *195*, 283.
4. Bush, C. E.; DiMichele, L. J.; Peterson, W. R.; Sherman, D. G.; Godsey, J. H. *Anal. Biochem.* **1992**, *202*, 146.
5. Seveus, L.; Väisälä, M.; Syrjänen, S.; Sandberg, M.; Kuusisto, A.; Harju, R.; Salo, J.; Hemmilä, I.; Kojola, H.; Soini, E. *Cytometry* **1992**, *13*, 329.
6. Morrow, J. R.; Buttrey, L. A.; Shelton, V. M.; Berback, K. A. *J. Am. Chem. Soc.* **1992**, *114*, 1903.
7. Komiyama, M.; Matsumoto, Y.; Matsumura K. *J. Chem. Soc. Chem. Commun.* **1992**, 640.
8. Kuusela, S.; Lönnberg, H. *J. Phys. Org. Chem.* **1993**, *6*, 347.
9. Lauffer, R. B. *Chem. Rev.* **1987**, *87*, 901.
10. Richardson, F. S. *Chem. Rev.* **1982**, *82*, 541.
11. Silverstein, R. M.; Bassler, C. G.; Morrill, T. C. "*Spectrometric Identification of Organic Compounds*," 3rd ed., Wiley, NY 1974, pp. 194-196.
12. Whan, R. E.; Crosby, G. A. *J. Mol. Spectrosc.* **1966**, *1*, 37.
13. Soini, E.; Hemmilä, I. *Clin. Chem.* **1979**, *25*, 353.
14. Lopez, E.; Chypre, C.; Alpha, B.; Mathis, G. *Clin. Chem.* **1993**, *39*, 196.
15. Mukkala, V.-M.; Sund, C.; Kwiatkowski, M.; Pasanen, P.; Högberg, M.; Kankare, J.; Takalo, H. *Helv. Chim. Acta* **1992**, *75*, 1621.
16. Mukkala, V.-M.; Kankare, J. *Helv. Chim. Acta* **1992**, *75*, 1578.
17. Diamandis, E. P.; Morton, R. C. *J. Immunol. Methods* **1988**, *112*, 43.
18. Reichstein, E.; Shami, Y.; Ramjeesingh, M.; Diamandis, E. P. *Anal. Chem.* **1988**, *50*, 1069.
19. Evangelista, R. A.; Pollak, A.; Allore, B.; Templeton, E. F.; Morton, R. C.; Diamandis, E. P. *Clin. Biochem.* **1988**, *21*, 173.
20. Takalo, H.; Mukkala, V.-M. **1991**, *PCT Appl./FI91/00373.*
21. Mukkala, V.-M.; Kwiatkowski, M.; Kankare, J.; Takalo, H. *Helv. Chim. Acta* **1993**, *76*, 893.
22. Alpha, B.; Lehn, J.-M.; Mathis, G. *Angew. Chem. Int. Ed.* **1987**, *26*, 266.
23. Sabbatini, N.; Perathoner, S.; Balzani, V.; Alpha, B.; Lehn, J.-M. in "*Supramolecular Photochemistry*", Balzani, V., ed., D. Reidel Publishing Company, 1987, pp. 187-206.
24. Balzini, V.; Lehn, J.-M.; van der Loosdrecht, J.; Mecati, A.; Sabbatini, N.; Zissel, R. *Angew. Chem.* **1991**, *30*, 190.
25. Lehn, J.-M.; Roth, C. O. *Helv. Chim. Acta* **1991**, *74*, 572.
26. Mukkala, V.-M.; Helenius, M.; Hemmilä, I.; Kankare, J.; Takalo, H., submitted to *Helv. Chim. Acta*.

27. Wieder, I.; Hidgson, K.O. **1977**, *Ger. Pat.* 2,628,158.
28. Salama, S.; Richardson, F. S. *Inorg. Chem.* **1990**, *19*, 635.
29. Bailey, M. P.; Rocks, B. F.; Riley, C. *Analyst* **1984**, *109*, 1449.
30. Sabbatini, N.; Guardigli, M.; Balzani, V.; Ghidini, E.; Pochini, A.; Ungaro, R. *Proceedings of Interdisciplinary Meeting on Luminescence: Fundamentals and Applications*, Bologna 1989, p. 40.
31. Bünzli, J.-C. G.; Froidevaux, P.; Besancon, F.; Harrowfield, J. M. *Proceedings of 29th International Conference on Coordination Chemistry*, Lausanne 1992, p. 39.
32. Kuusela, S.; Lönnberg, H. *J. Phys. Org. Chem.* **1992**, *5*, 803.
33. Sumaoka, J.; Yashiro, M.; Komiyama, M. *J. Chem. Soc. Chem. Commun.* **1992**, 1707.
34. Breslow, R.; Huang, D.-L. *Proc. Natl. Acad. Sci. USA* **1991**, *88*, 4080.
35. Järvinen, P.; Oivanen, M.; Lönnberg, H. *J. Org. Chem.* **1991**, *56*, 5396.
36. Kuusela, S.; Lönnberg, H. unpublished results.
37. Beaucage, S. L.; Iyer, R. P. *Tetrahedron* **1993**, *49*, 1925.
38. Hovinen, J.; Gouzaev, A.; Azhayev, A.; Lönnberg, H. *Tetrahedron Lett.* in press.
39. Azhayev, A.; Gouzaev, A.; Hovinen, J.; Azhayeva, E.; Lönnberg, H. *Tetrahedron Lett.* in press.

**Received: August 12, 1993**

